# ‘*Candidatus* Liberibacter asiaticus’ Effector SDE525 hijacks NACα to Suppress Jasmonic Acid‐Mediated Immunity in Citrus

**DOI:** 10.1111/mpp.70272

**Published:** 2026-05-18

**Authors:** Jiefu Deng, Yijie Zhan, Zixiang Sun, Changwei Luo, Na Song, Jinxing Liao, Zehua Zhou, Tuyong Yi

**Affiliations:** ^1^ College of Plant Protection Hunan Agricultural University/Hunan Provincial Key Laboratory for Biology and Control of Plant Pests Changsha Hunan China; ^2^ State Key Laboratory of Hybrid Rice Hunan Hybrid Rice Research Center Changsha China

**Keywords:** ‘*Candidatus* Liberibacter asiaticus’, citrus huanglongbing, jasmonic acid, NACα, secretory property

## Abstract

Citrus huanglongbing (HLB), caused by the phloem‐limited bacterium ‘*Candidatus* Liberibacter asiaticus’ (*C*Las), is one of the most destructive diseases in global citrus production. Here, we report the functional characterization of a core *C*Las effector, CLIBASIA_00525 named SDE525, that manipulates host immunity by targeting the citrus nascent polypeptide‐associated complex alpha subunit (CsNACα). We confirmed that SDE525 is a secreted protein that localizes to the nucleus, cytoplasm, and plasma membrane. Notably, the effect of SDE525 exhibits host‐specific context dependence: while its transient expression in the non‐host *Nicotiana benthamiana* unexpectedly triggered immune responses, in its native host, sweet orange ( *Citrus sinensis* ), SDE525 interacted with CsNACα to modulate defence, as demonstrated by multiple independent assays. Integrated transcriptomic and metabolomic analyses revealed that this interaction reprograms the host's hormonal landscape, suppressing the jasmonic acid (JA) signalling pathway while simultaneously activating the salicylic acid (SA) pathway. Importantly, under transient overexpression conditions in citrus, this immune reprogramming enhanced resistance against the bacterial canker pathogen *Xanthomonas citri* subsp. *citri*—a phenomenon distinct from the susceptibility observed during authentic *C*Las infection. Our findings uncover a sophisticated mechanism by which a *C*Las effector rewires host defence signalling, providing fundamental insights into its pathogenesis with potential long‐term implications for future HLB management.

## Graphical abstract

The ‘*Candidatus* Liberibacter asiaticus’ secreted protein SDE525 suppresses the plant jasmonic acid defence pathway by targeting the citrus NACα protein, thereby weakening citrus resistance against the Asian citrus psyllid.

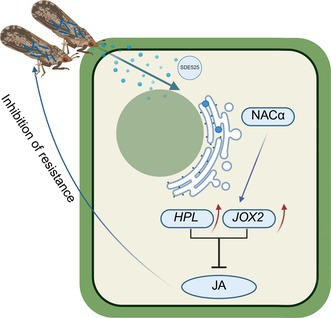

## 1 Introduction

Citrus huanglongbing (HLB), associated with the phloem‐limited bacterium ‘*Candidatus* Liberibacter asiaticus’ (*C*Las), is the most devastating disease threatening citrus production worldwide (Ma et al. 2022). The pathogen is efficiently transmitted by the Asian citrus psyllid (ACP, *Diaphorina citri* ), with infected adults capable of spreading *C*Las for their entire lifespan (Nian et al. 2024; He et al. 2023). To date, the unculturable nature of *C*Las has significantly hindered research into its pathogenesis. Consequently, a primary strategy to unravel the molecular mechanisms of HLB has been to identify and functionally characterize the Sec‐dependent effectors (SDEs) that *C*Las secretes into the host plant (Shen et al. 2022). While the direct causative agents of HLB symptoms, such as leaf mottling and fruit deformity, remain elusive, it is widely accepted that *C*Las effectors play a pivotal role in manipulating host physiology and suppressing immunity to facilitate infection (Ma et al. 2022).

Plants possess a sophisticated, layered innate immune system. The first layer, PAMP‐triggered immunity (PTI), is activated when cell surface receptors detect conserved pathogen‐associated molecular patterns (PAMPs) (Wang et al. 2022). In a classic evolutionary arms race, successful pathogens deploy a suite of effector proteins into host cells to suppress PTI and reprogramme host physiology, leading to effector‐triggered susceptibility (ETS). A second layer of defence, effector‐triggered immunity (ETI), is activated when intracellular resistance (R) proteins directly or indirectly recognize specific effectors, often resulting in a robust and rapid immune response (Shi et al. 2019; Pitino et al. 2016). To date, research on *C*Las effectors has revealed several key virulence strategies, broadly categorized as follows (Cui et al. 2023): (a) modulation of host cell death, where effectors like SDE1 can trigger cell death in *Nicotiana benthamiana* (Pitino et al. 2016), while others like AGH17470 induce a hypersensitive response (HR) (Du et al. 2022); (b) direct interaction with host factors to suppress defence, such as SDE1 targeting citrus papain‐like cysteine proteases (PLCPs) (Clark et al. 2018) and AGH17488 enhancing host susceptibility by targeting Ascorbate Peroxidase 6 (APX6) (Du et al. 2023); and (c) manipulation of host autophagy, exemplified by SDE3 and SDE4405, which interact with host proteins to modulate autophagic pathways (Shi, Gong, et al. 2023; Shi, Yang, et al. 2023). These findings underscore the diverse tactics employed by *C*Las to subvert plant immunity. However, beyond targeting dedicated immune regulators, an emerging paradigm in plant pathology is that pathogens frequently hijack fundamental cellular machinery—such as the translation and protein‐folding apparatus—to induce broad physiological reprogramming. Consequently, ribosome‐associated chaperones represent highly attractive, yet largely underexplored, hubs for effector‐mediated sabotage.

The nascent polypeptide‐associated complex (NAC) is a highly conserved ribosome‐associated chaperone that binds nascent polypeptides as they emerge from the ribosomal tunnel exit (Jomaa et al. 2022). By competing with the signal recognition particle (SRP), NAC prevents the mistargeting of nascent chains to the endoplasmic reticulum (Beatrix et al. 2000). While the structural and biochemical functions of NAC have been extensively characterized in yeast and animals (Rospert et al. 2002; Panasenko et al. 2006), its specific roles in plant biology are only beginning to be delineated. Early studies indicated its involvement in development, such as the abnormal leaf morphology observed when NACβ is silenced in *N. benthamiana* (Bloss et al. 2003). More recently, NACα has been linked to plant–pathogen interactions, facilitating the cell‐to‐cell movement of brome mosaic virus (BMV) (Fíla et al. 2020). Despite these established roles in basic cellular physiology, the potential of NAC subunits being directly targeted by plant pathogen effectors to modulate immunity remains a critical knowledge gap.

To address this gap, our study focused on SDE525 (CLIBASIA_00525), a core effector highly conserved across diverse *C*Las strains (Thapa et al. 2020). Given the fundamental role of NAC in protein homeostasis, we hypothesized that *C*Las might exploit this complex. We demonstrate that SDE525 is secreted into the host cell, where it directly targets the citrus NACα (CsNACα). Subsequent integrated multi‐omics analyses revealed that this specific interaction acts as a molecular switch to reprogramme host hormonal signalling, suppressing the jasmonic acid (JA) pathway while activating the salicylic acid (SA) pathway.

Crucially, this immune reprogramming creates a dual phenotype: while it facilitates the acquisition and transmission of *C*Las by its psyllid vector, it paradoxically enhances resistance to the pathogen *Xanthomonas citri* . Collectively, our work uncovers a sophisticated virulence mechanism in which a bacterial pathogen hijacks a core component of the host's protein translation machinery to subvert immunity. This not only provides a new dimension to our understanding of *C*Las pathogenesis but also reveals the SDE525‐CsNACα module as a potential Achilles' heel for developing targeted disease control strategies.

## 2 Results

### 2.1 CLIBASIA_00525 Is a Conserved Secreted Effector With Diverse Subcellular Localizations in *N. benthamiana*

**FIGURE 1:**
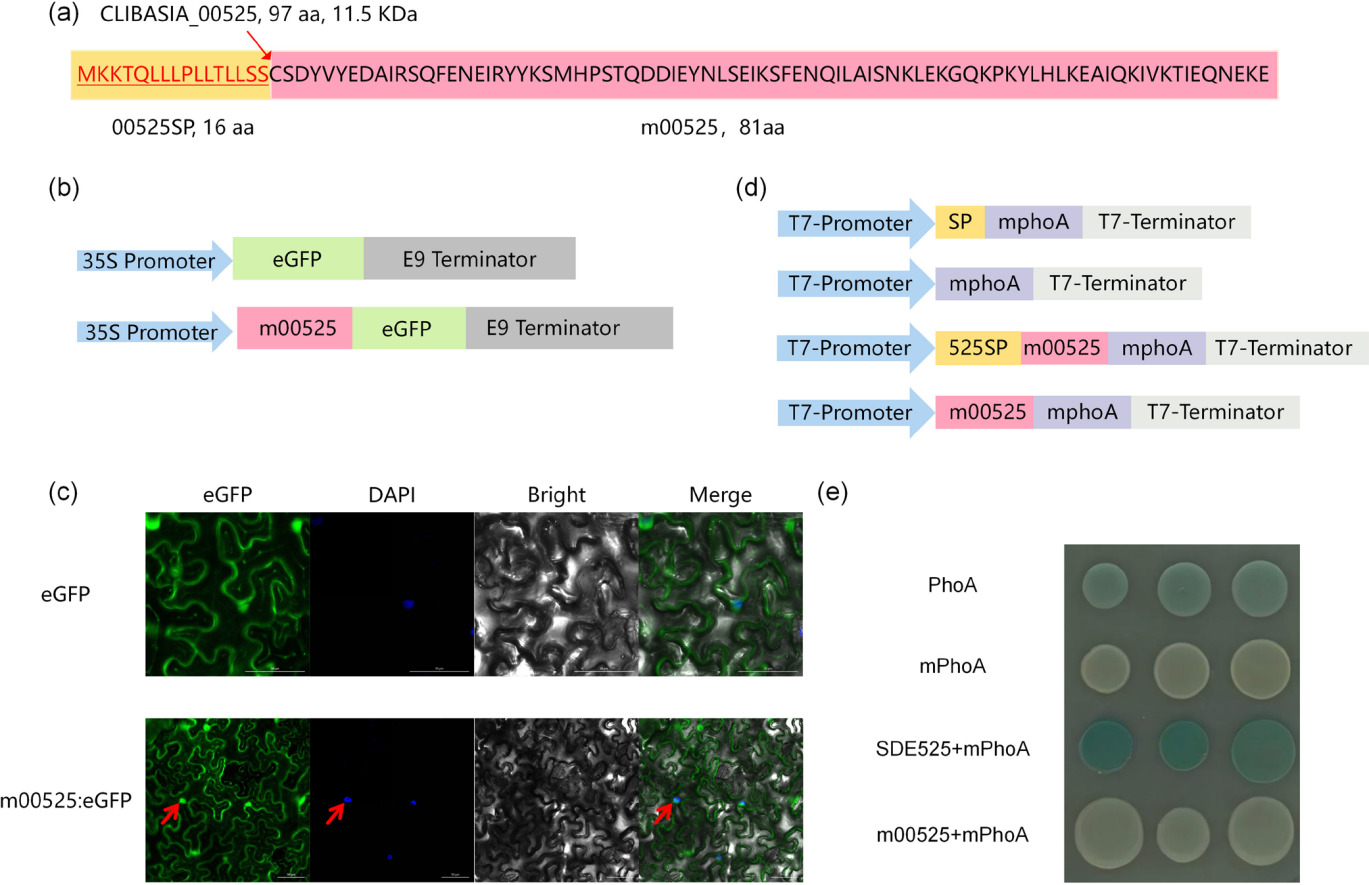
Subcellular localization and secretory verification of SDE525. (a) Analysis of SDE525 amino acid sequence. Amino acid sequence of SDE525 (97 amino acids, 11.5 kDa) with N‐terminal signal peptide (00525SP, highlight in yellow), red arrow indicates the cleavage site between 16 and 17 amino acid positions (LSS‐CS). (b) The schematic diagram of expression construct for subcellular localization experiments. (c) M00525 was localized in multiple subcellular compartments of *Nicotiana benthamiana*. Subcellular localization of the m00525:EGFP fusion protein was observed via confocal microscopy. Nuclei were counterstained with DAPI to confirm nuclear localization. Imaging was performed with the following parameters: eGFP (488 nm excitation, 510 nm emission) and DAPI (405 nm excitation, 450 nm emission). Scale bars represent 50 μm. (d) The schematic diagram of the expression construct for secretion validation experiments. (e) SDE525 directed the extracytoplasmic secretion of mPhoA. *Escherichia coli* cells expressing the fusion proteins SDE525‐mPhoA were incubated at 37°C in indicator Luria Bertani medium that contained Na_2_HPO_4_ (75 mM), isopropyl‐β‐D‐thiogalactopyranoside (IPTG) (100 mM) and 5‐bromo‐4‐chloro‐3‐indolyl phosphate p‐toluidine salt (BCIP) (90 μg/mL). The image was taken at 16 h of incubation.

Bioinformatic analysis of CLIBASIA_00525 (SDE525) revealed a 97‐amino‐acid (aa) protein with a predicted N‐terminal 16‐aa signal peptide (00525SP; Figure 1a) and no transmembrane domains, classifying it as a secreted effector. To investigate the subcellular localization of mature SDE525 (m00525) in plant cells, a C‐terminal fusion with enhanced green fluorescent protein (eGFP) was constructed, with expression driven by the CaMV 35S promoter (Figure 1b). Confocal microscopy of *N. benthamiana* leaves transiently expressing the fusion protein revealed that m00525‐eGFP fluorescence was distributed throughout both the cytosol and the nucleus. To definitively confirm the nuclear accumulation, the infiltrated leaf tissues were simultaneously stained with DAPI, a fluorescent dye that specifically binds to DNA. As shown in Figure 1c, the green m00525‐eGFP signal exhibited a clear overlap with the blue DAPI fluorescence within the nuclei. This distribution pattern was highly similar to that of the free eGFP control and was further validated by co‐localization with specific nuclear and plasma membrane markers (Figure 1c).

To validate the secretion capability of SDE525, an alkaline phosphatase (PhoA) assay was conducted in *Escherichia coli* . The principle of this assay is that secretion of a PhoA fusion protein into the periplasm, where PhoA becomes enzymatically active, leads to the development of blue colonies on indicator plates (Figure 1d). Consistent with this principle, colonies expressing the SDE525‐mPhoA fusion exhibited blue colouration, whereas colonies expressing the mPhoA control remained colourless (Figure 1e). This finding confirms that the N‐terminal signal peptide of SDE525 is functional, mediating protein secretion.

### 2.2 SDE525 Expression Triggers Defence Responses in *N. benthamiana*

**FIGURE 2:**
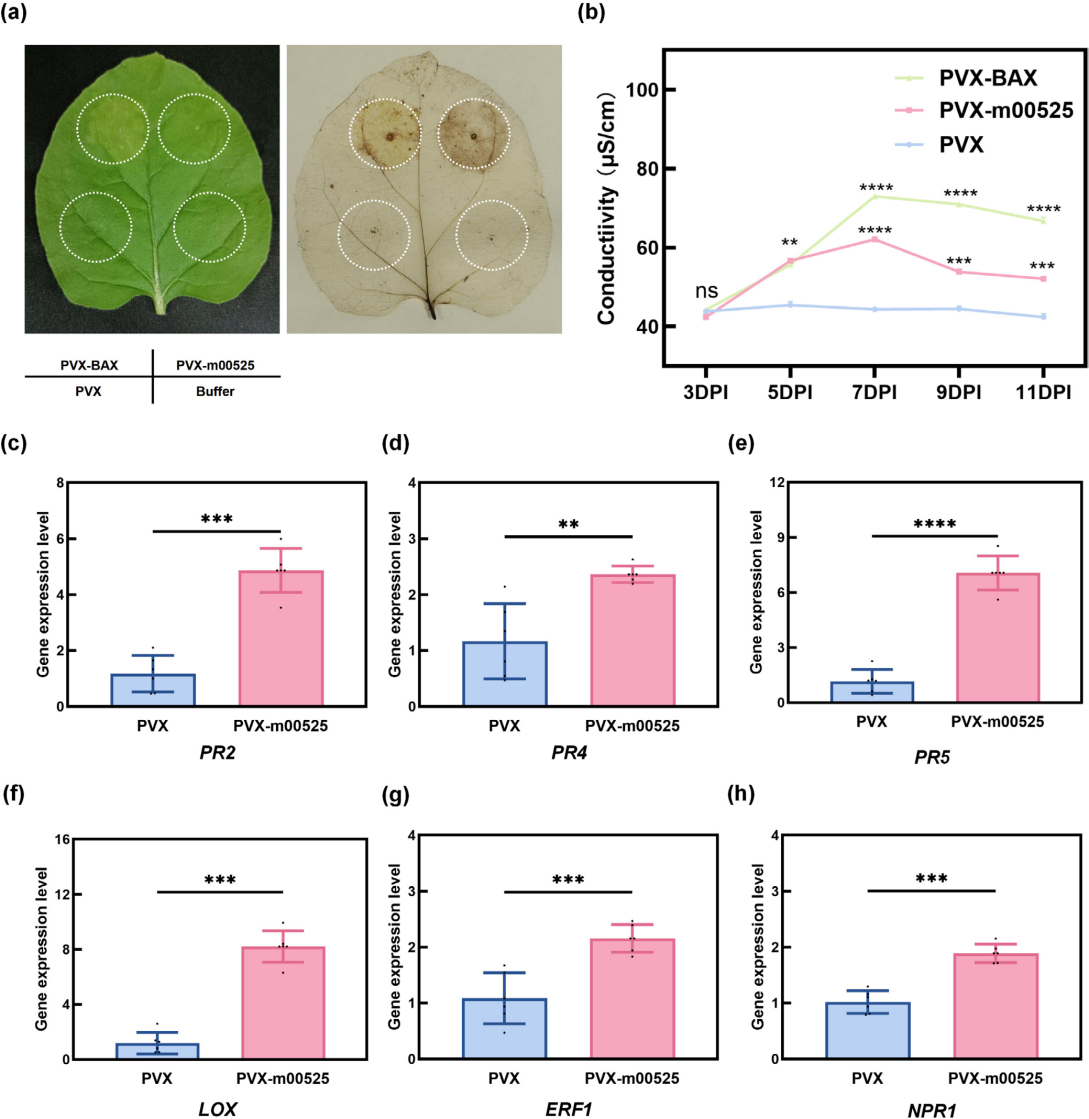
SDE525 in *Nicotiana benthamiana* causes the reactive oxygen species (ROS) burst, ion leakage and expression of genes related to defence response. (a) Symptoms on leaf infiltrated with PVX, PVX‐m00525, PVX‐BAX and buffer at 3 days post‐inoculation (dpi), and leaf stained with 3,3′‐diaminobenzidine to visualize ROS. The experiment was repeated three times with at least six leaves each time. (b) Electrolyte leakage induced by SDE525 post‐agroinfiltration. The conductivity of the solution was measured after 1 h of shaking. Error bars indicate standard errors. The experiment had three biological replicates and three technical replicates. Data are expressed as the mean ± SE (*n* = 3), and asterisks indicate significant differences (Student's *t* test, *p < 0.05, **p < 0.01, ***p < 0.001, ****p < 0.0001). (c–h) The experiment comprised at least three independent biological replicates and three technical replicates. Data are presented as mean ± SD (*n* = 6), and asterisks indicate significant differences (Student's *t* test, *p < 0.05, **p < 0.01, ***p < 0.001, ****p < 0.0001).

To investigate the potential role of SDE525 in pathogenicity, the effector was transiently expressed in *N. benthamiana* using a potato virus X (PVX)‐based system. Infiltration with PVX‐m00525 or the positive control PVX‐BAX resulted in clear necrotic lesions, whereas infiltration with the negative controls (empty PVX vector or buffer) produced no visible symptoms (Figure 2a). This visible cell death was quantified by measuring electrolyte leakage, which was significantly higher in *SDE525*‐expressing leaves compared to leaves infiltrated with the empty PVX vector at 7 days post‐infiltration (dpi) (Figure 2b). To further characterize this defence response at the molecular level, the transcriptional levels of key defence marker genes were evaluated by reverse transcription‐quantitative PCR (RT‐qPCR). The expression of the pathogenesis‐related genes *NbPR2* and *NbPR5*, as well as the lipoxygenase gene *NbLOX*, was strongly induced in *SDE525*‐expressing leaves relative to the PVX control (Figure 2c–h). Collectively, these molecular markers, together with the phenotypic and physiological data, demonstrate that SDE525 strongly induces defence‐associated responses in *N. benthamiana*. However, it is crucial to distinguish this apparent elicitor‐like activity observed in a heterologous system from its actual virulence function in its natural host. As our subsequent data in citrus reveal, rather than triggering generalized cell death, SDE525 acts as a sophisticated virulence factor that subtly reprogrammes host immunity, specifically by suppressing JA signalling to favour the pathogen's lifecycle.

### 2.3 SDE525 Interacts With the *C. sinensis* NACα

To identify the host targets of SDE525, a co‐immunoprecipitation (Co‐IP) assay was performed and coupled with mass spectrometry (MS). An eGFP‐tagged version of SDE525 (m00525‐eGFP) was transiently expressed in *Citrus sinensis* leaves via *Agrobacterium tumefaciens* infiltration. Total proteins were extracted from the infiltrated tissue, and the m00525‐eGFP protein complex was immunoprecipitated with anti‐GFP magnetic beads. After stringent washing, the bound proteins were eluted and separated by SDS‐PAGE. The presence of the target protein was confirmed by western blotting using an anti‐GFP antibody (Figure S1), and the co‐precipitated host proteins were then identified by LC–MS/MS. The resulting peptide spectra were searched against the *C. sinensis* UniProt database (Table 1). The MS data were screened to identify proteins involved in the defence response and its regulation, which were subsequently subjected to a yeast two‐hybrid (Y2H) assay to validate direct binary interactions with SDE525. Among the tested candidates, only the nascent polypeptide‐associated complex alpha subunit (CsNACα) yielded a positive interaction, thereby emerging as a specific, high‐confidence interactor. CsNACα is a component of the evolutionarily conserved NAC complex that binds nascent polypeptides at the ribosomal tunnel exit. It plays a critical role in protein quality control by competing with the signal recognition particle (SRP) to prevent the mistargeting of cytosolic proteins to the endoplasmic reticulum (ER) (Jomaa et al. 2022; Yamada et al. 2023).

**TABLE 1:** Selected citrus protein targets of *C*Las m00525.

Protein name and ID	Protein score	Function
A0A067D4M8 KOW domain‐containing protein	2420	The primary role of KOW domains is RNA binding, which is mediated by the domain's β‐barrel structure and conserved residues. This function is critical for transcription regulation/ribosomal biogenesis/RNA processing and degradation (Wang et al. 2025).
A0A067FYR7 Histone H2A	2343.2	Histone H2A's primary role is in chromatin organization: it packages DNA into nucleosomes, enabling efficient storage of genetic material while regulating access to transcriptional machinery (Yin et al. 2024).
A0A067DGW1 NAC‐A/B domain‐containing protein	1609.4	The NAC‐A/B domain is central to the functions of NAC proteins, enabling them to perform diverse roles in cellular homeostasis: DNA binding and transcriptional regulation, protein–protein interactions, chaperone function (NAC complex) (Ding et al. 2018)
A0A067F9W2 Tubulin β chain	1273.7	TUBB's primary role is in MT‐dependent processes: cell division, intracellular transport, cytoskeletal organization, specialized roles (Liu et al. 2018).
A0A067EVM6 UspA domain‐containing protein	740.6	UspA proteins are stress responders that enhance cell survival during prolonged exposure to adverse conditions (Sinha et al. 2016).
A0A067DQU7 Embryo‐specific protein 3	539.4	Embryo‐specific proteins are a class of proteins whose expression is spatially or temporally restricted to specific stages or cell types during embryonic development (Restovic et al. 2017).
A0A067FNN3 2‐oxoglutarate dehydrogenase, mitochondrial	488.1	OGDH catalyses the irreversible oxidative decarboxylation of 2‐oxoglutarate (α‐ketoglutarate) to form succinyl‐CoA and carbon dioxide (CO₂) (Nemeria et al. 2018).
A0A067EEM8 Translation machinery‐associated protein 22	480.6	As a putative translation initiation factor, TMAP22 is hypothesized to participate in the early stages of translation by facilitating the binding of mRNA to the small (40S) ribosomal subunit or stabilizing the pre‐initiation complex. This function is essential for ensuring accurate and efficient protein synthesis, which is fundamental for cellular growth, development, and stress responses in plants (Ma et al. 2025).
A0A067E2B3 Alcohol dehydrogenase‐like C‐terminal domain‐containing protein	428.1	The primary function of these proteins is catalysing redox reactions involving alcohols, aldehydes, and ketones. Specific roles vary by organism and isoform: plant metabolism, mammalian detoxification, bacterial stress response (Klein et al. 2024).
A0A067EAK1 S‐formylglutathione hydrolase	377.9	SFGH is integral to the formaldehyde detoxification pathway, which operates in concert with formaldehyde dehydrogenase (FALDH, S‐hydroxymethylglutathione dehydrogenase) (Kordic et al. 2002).
A0A067H184 AAA+ ATPase domain‐containing protein	345	AAA+ ATPase domain‐containing proteins are involved in a wide range of cellular processes, including protein quality control, proteolysis, dna replication/repair, membrane dynamics, cytoskeletal regulation (Goel and Kumar 2024).

**FIGURE 3:**
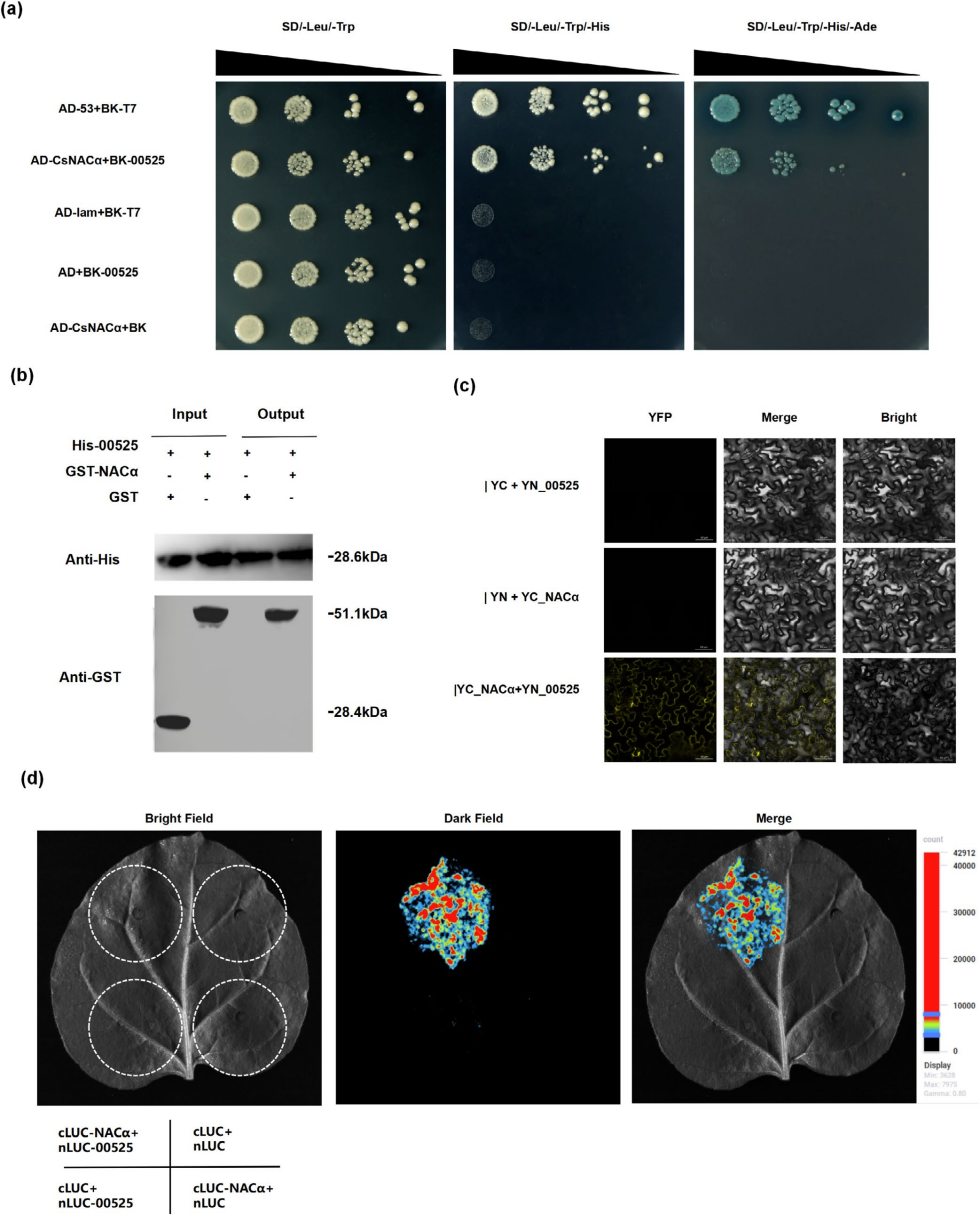
SDE525 interacts with NACα protein in *Citrus sinensis* . (a) Verification of the interaction of SDE525 with CsNACα by yeast two‐hybrid (Y2H) assay. (b) In vitro His pull‐down assay to detect direct interaction between SDE525 and CsNAC. His6‐SDE52 and GST CsNACα were incubated with His beads. The input and pull‐down proteins were detected with anti‐His and anti‐GST antibodies. (c) Bimolecular fluorescence complementation (BiFC) assay showed the interaction between SED525 and CsNACα in vivo. (d) Luciferase complementation (LCI) assay showed the interaction between SDE525 and CsNACα in vivo.

To validate this interaction, a series of independent biochemical and cellular assays was employed. Y2H analysis demonstrated a direct interaction between SDE525 and CsNACα in yeast cells (Figure 3a). This finding was further corroborated by an in vitro pull‐down assay, which confirmed the physical binding between the purified proteins (Figure 3b). Moreover, the interaction was recapitulated in planta, as evidenced by both the reconstitution of YFP fluorescence in a bimolecular fluorescence complementation (BiFC) assay (Figure 3c) and a significant increase in luciferase activity in a luciferase complementation imaging (LCI) assay (Figure 3d). Collectively, these results provide robust evidence that SDE525 directly interacts with the citrus CsNACα protein.

To determine the subcellular site of the interaction between SDE525 and NACα, co‐localization analysis was performed in *N. benthamiana* leaves. For this analysis, NACα was fused to eGFP (NACα‐eGFP), while m00525 was fused to mCherry (m00525‐mCherry). Consistent with our previous findings, m00525‐mCherry exhibited a dispersed signal throughout the cytosol and nucleus. Notably, NACα‐eGFP displayed a highly similar, multicompartmental localization pattern. Analysis of the merged images revealed significant overlap between the m00525‐mCherry and NACα‐eGFP signals, confirming their co‐localization. Furthermore, the expression of m00525 did not alter the native subcellular distribution of NACα, suggesting that the effector associates with NACα at its physiological locations without causing its mislocalization (Figure S2).

### 2.4 Transient Expression of SDE525 and *CsNACα* in *C. sinensis* Enhances Resistance to *X. citri* subsp. *citri*

**FIGURE 4:**
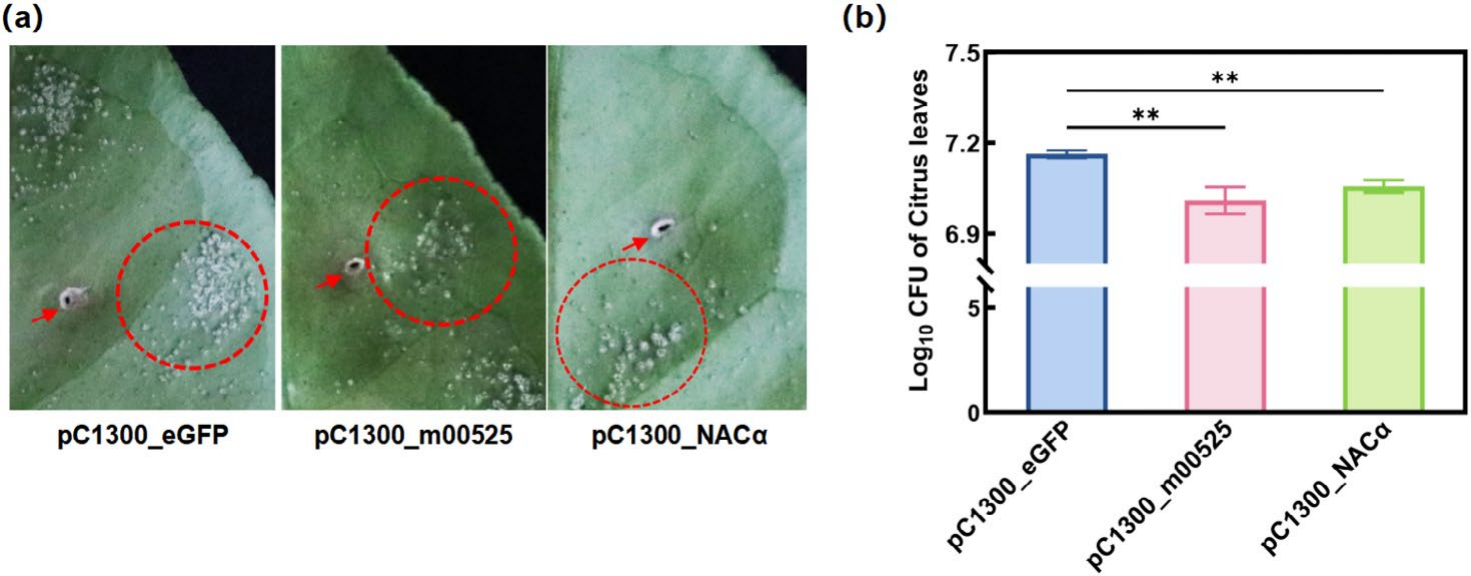
Evaluation of susceptibility to *Xanthomonas citri* subsp. *citri* by transient expression of m00525 and NACα in citrus. (a) Red circles indicate the necrotic lesions, while red arrows mark the infiltration sites. (b) Colony‐forming units (CFU) from symptomatic leaves were counted to assess the accumulation of the bacterial population. **p < 0.01, significant difference determined by Student's *t* test. Data are expressed as the mean ± SE (*n* = 3).

*Xanthomonas citri* subsp. *citri* is a gram‐negative bacterium responsible for citrus bacterial canker, which is another devastating citrus bacterial disease worldwide. To assess the role of SDE525 in resistance to citrus bacterial canker, the effector m00525 was transiently expressed in citrus leaves, which were then challenged with *X. citri* via fine‐needle inoculation. Leaves transiently expressing m00525 developed significantly smaller canker lesions and supported lower bacterial growth compared to control leaves (Figure 4). Collectively, these results demonstrate that transient expression of m00525 enhances resistance to *X. citri* by reducing disease symptom development and bacterial proliferation in citrus leaves.

### 2.5 SDE525‐CsNACα Interaction Reprogrammes JA/SA Signalling

RNA sequencing (RNA‐Seq) was employed to investigate changes in gene expression in *C. sinensis* leaves in response to the transient expression of SDE525 and CsNACα. Leaf samples were collected from three treatment groups: an eGFP‐expressing control (Control), an eGFP+m00525 co‐expression group (m00525), and an eGFP+NACα co‐expression group (NACα). The sequenced reads were processed bioinformatically to elucidate the molecular mechanisms influenced by m00525 and NACα in citrus. In total, across all nine sequenced samples, 371,114,244 Gb of raw data were obtained (Table S3), resulting in approximately 369,973,672 clean reads after filtration. The G + C content across all samples ranged from 43.51% to 44.33% (Table S3), and the average percentage of high‐quality reads (Q ≥ 30) was 94.94% (Table S3). The percentage of uniquely mapped reads varied from 80.45% to 87.53% (Table S3). According to the mapping results, a total of 19,426 genes were mapped in the genome. Overall, this transcriptome sequencing experiment profiled transcriptomic changes in citrus under transient expression of different genes.

**FIGURE 5:**
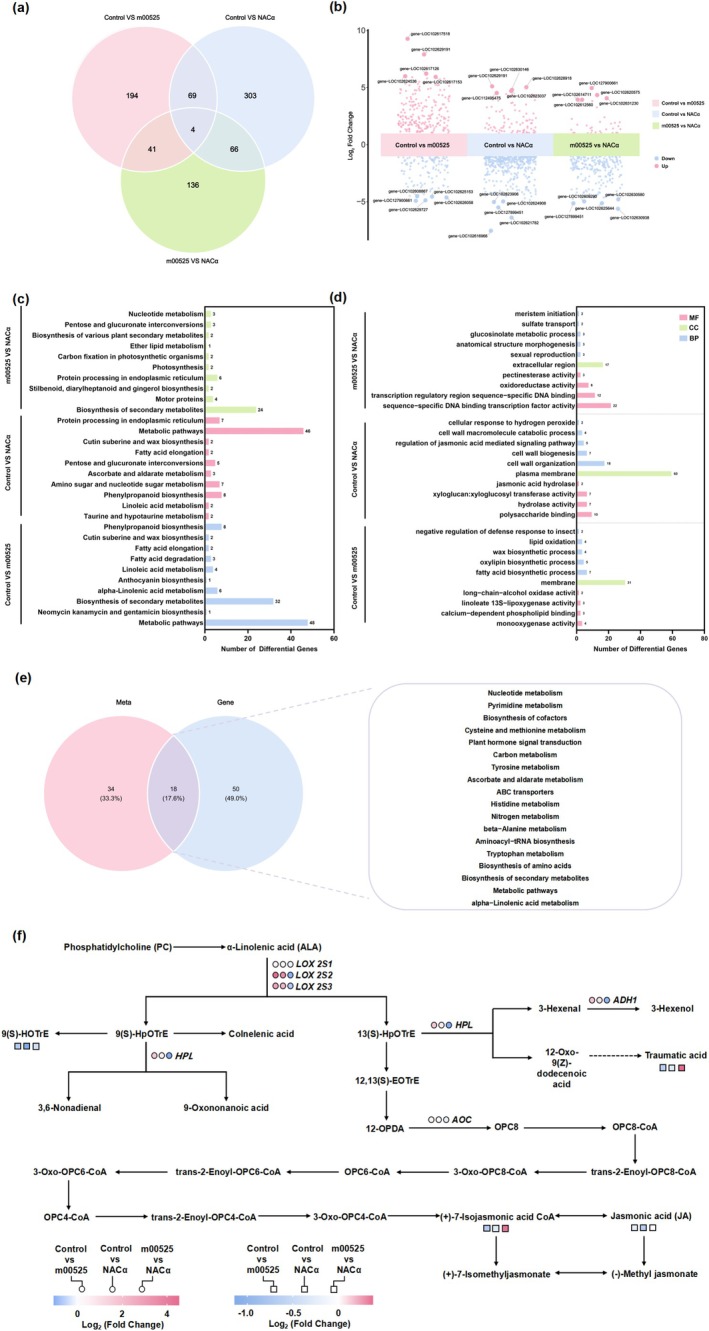
Transcriptome data and metabolites analysis of different gene transient expression. (a) Venn graph showing overlapping differentially expressed genes (DEGs) (and up‐ and down‐regulated genes) among the three groups. A total of 813 DEGs between gene transient expression groups and Control. (b) DEG analysis showing up‐ and down‐regulated genes across all three comparison groups. A log_2_FC > 1 and *p* < 0.01 is indicated as significantly up‐regulated in red, while a log_2_FC < −1 and *p* < 0.01 is indicated as significantly down‐regulated in blue. (c) Control versus m00525, Control versus NACα, m00525 versus NACα DEGs were annotated to the KEGG database. (d) Control versus m00525, Control versus NACα, m00525 versus NACα DEGs are annotated to the GO database. (e) The Venn diagram illustrates the distribution of DEGs and differentially accumulated metabolites (DAMs) in the Control versus m00525 comparison. Pathway enrichment analysis using KEGG revealed 18 shared pathways significantly enriched by both DEGs and DAMs. (f) Based on KEGG enrichment analysis, the linolenic acid metabolism pathway (KO00592) was mapped to visualize the signalling pathway, with DEGs and DAMs annotated on the pathway map.

Differentially expressed genes (DEGs) were identified through the three comparison groups: Control versus m00525, Control versus NACα, and m00525 versus NACα (Figure S3). In the Control versus m00525 comparison, 308 DEGs were identified (202 up‐ and 106 down‐regulated). The Control versus NACα comparison yielded 442 genes (82 up‐ and 360 down‐regulated), while the m00525 versus NACα comparison revealed 247 DEGs (36 up‐ and 211 down‐regulated) (Table S4). A Venn diagram analysis was performed to identify shared and unique DEGs among the three groups. A total of 73 DEGs were common to both the m00525 and NACα expression groups when compared to the control. Notably, four DEGs showed consistent differential expression across all transient dose comparisons (Figure 5a). A more in‐depth examination of DEGs revealed significant variations in the magnitude of expression changes across the different treatments (Figure 5b). Kyoto Encyclopedia of Genes and Genomes (KEGG) enrichment analysis was conducted on the identified DEGs to elucidate their functional roles and involvement in biological pathways (Estrella‐M et al.2023). KEGG pathway enrichment analysis revealed that the DEGs induced by m00525 and NACα were significantly enriched in ‘Metabolic pathways’, ‘Biosynthesis of secondary metabolites’, and ‘Phenylpropanoid biosynthesis’ (Figure 5c). Gene ontology (GO) annotation further revealed the roles of these DEGs within three main categories: Biological Process (e.g., regulation of the JA‐mediated signalling pathway, cell wall modification), Cellular Component (e.g., plasma membrane, extracellular region), and Molecular Function (e.g., sequence‐specific DNA binding transcription factor activity) (Figure 5d, Table S5).

A metabolomics analysis was performed to identify alterations in citrus leaf metabolites induced by different gene transient expression. Ultra‐performance liquid chromatography–tandem mass spectrometry (UPLC‐MS/MS) was used to profile metabolites and detect differentially accumulated metabolites (DAMs) in response to m00525 and NACα. This comprehensive metabolite analysis aimed to elucidate metabolic changes underlying *Citrus'* responses to m00525 and NACα. By comparing Control versus m00525, Control versus NACα, and m00525 versus NACα, DAMs were identified. Specifically, the Control versus m00525 comparison identified 127 DAMs (60 up‐regulated and 67 down‐regulated), the Control versus NACα comparison identified 246 DAMs (72 up‐regulated and 174 down‐regulated), and the m00525 versus NACα comparison identified 290 DAMs (70 up‐regulated and 220 down‐regulated) (Figure S4a–c).

The association between key DEGs and DAMs was evaluated using correlation analysis. KEGG pathway enrichment analysis identified 18 pathways in the Control versus m00525 comparison that were significantly enriched among both the DEGs and DAMs (Figure 5e). Similarly, 14 and 6 significantly enriched pathways were identified in the Control versus NACα and m00525 versus NACα comparisons, respectively (Figure S4d–f). A heatmap was generated to visualize the DEGs and DAMs that were specifically enriched in the α‐linolenic acid metabolism and phenylpropanoid biosynthesis pathways. Notably, α‐linolenic acid metabolism and phenylpropanoid biosynthesis are two significantly enriched metabolic pathways in both treatments (Figure S4g,h). Furthermore, a combined analysis of the transcriptome and metabolome data mapping to the α‐linolenic acid metabolism pathway (ko00592) was conducted and graphed (Figure 5f).

To validate the RNA‐Seq results, 10 DEGs exhibiting significant up‐ or down‐regulation were confirmed via reverse transcription‐quantitative PCR (RT‐qPCR). Additionally, to verify the metabolomics results, SA and JA levels were initially assessed by ELISA (Figure S5). To further strengthen this validation with a more robust and specific analytical approach, targeted HPLC was also employed to precisely quantify the levels of free SA, methyl salicylate (MeSA), JA, and methyl jasmonate (MeJA).

### 2.6 Targeted HPLC Quantification Validates SDE525‐Mediated SA/JA Reprogramming

**FIGURE 6:**
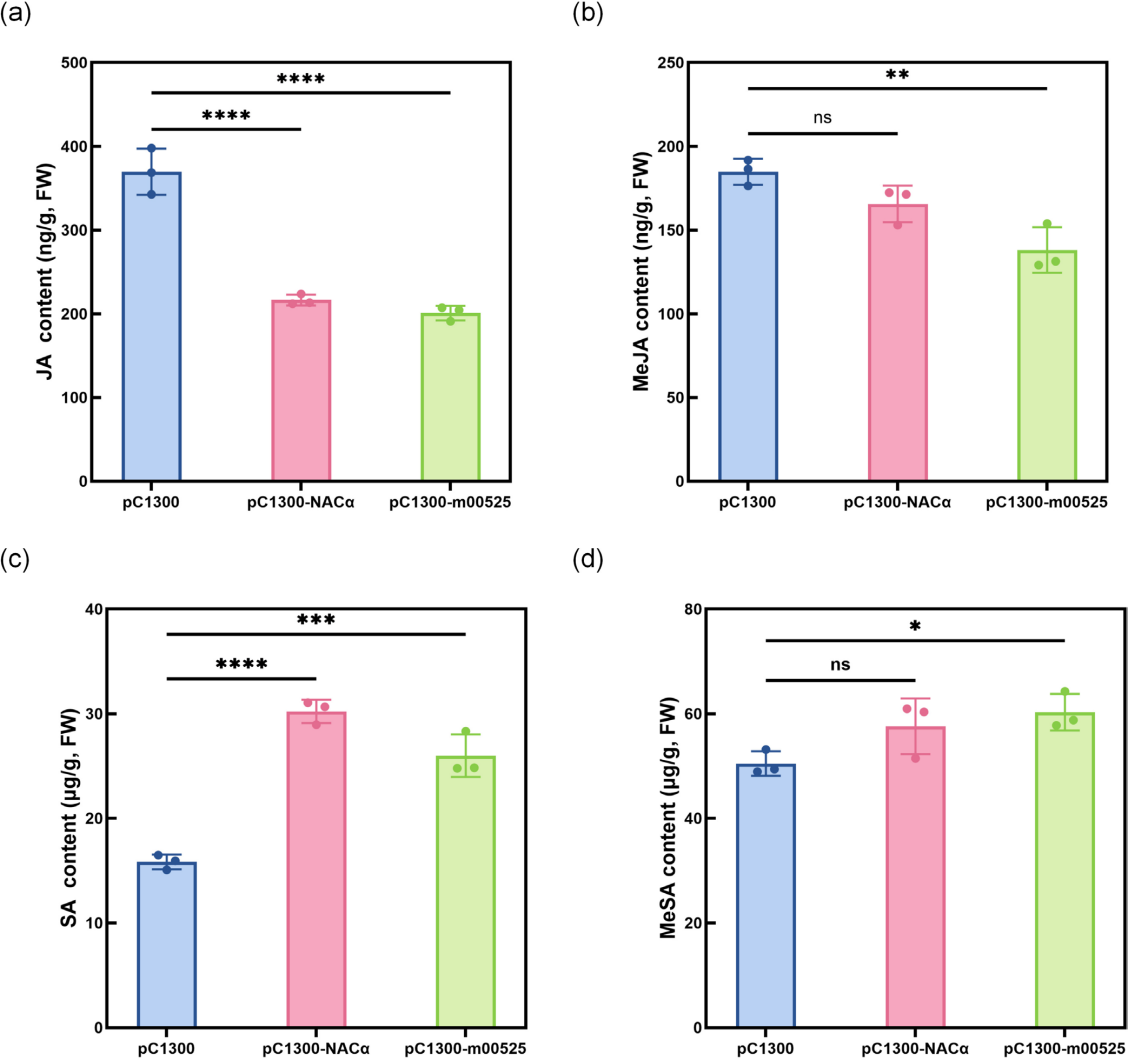
Dynamic changes in endogenous jasmonic acid (JA), methyl jasmonate (MeJA), salicylic acid (SA) and methyl salicylate (MeSA) contents in citrus leaves under different transient expression backgrounds. The endogenous levels of JA, MeJA, SA and MeSA in sweet orange leaves were quantified via HPLC at 72 h post‐inoculation across different treatments. Data are shown as mean ± SD (*n* = 3). Statistical significance relative to the respective mock control was assessed by a two‐tailed Student's *t* test (*p < 0.05, **p < 0.01, ***p < 0.001, ****p < 0.0001). FW, fresh weight.

To robustly validate the hormone reprogramming suggested by our untargeted metabolomics, we replaced the initial antibody‐based screening with a targeted, highly specific analytical approach. Specifically, we employed HPLC to precisely quantify the absolute concentrations of key signalling molecules: free SA, MeSA, free JA, and MeJA. This targeted HPLC analysis successfully resolved the critical limitation of distinguishing between the free acid forms and their corresponding methyl esters. Targeted quantification revealed distinct but interconnected hormonal profiles. Transient expression of *SDE525* significantly elevated both free SA and its methylated derivative (MeSA), while concomitantly causing a marked decrease in both free JA and MeJA (Figure 6). In contrast, while the manipulation of CsNACα alone similarly drove the core antagonistic shift—significantly increasing SA and decreasing JA—it did not significantly alter the levels of MeSA or MeJA (Figure 6). These precise quantitative profiles delineate a nuanced reprogramming mechanism: both SDE525 and its direct target CsNACα are sufficient to suppress JA and activate SA signalling, but the broader metabolic conversion into the mobile methylated hormone forms (MeSA and MeJA) appears to be specifically orchestrated by the SDE525 effector.

## 3 Discussion

Significant progress has been made in elucidating the functions of key genes in *C*Las, the pathogen responsible for HLB, particularly through the identification and functional characterization of its effector proteins (Hu et al. 2025). While the aetiology of HLB's characteristic symptoms—such as leaf mottling, corky veins, and fruit deformity—is complex and likely multifactorial (Ma et al. 2022), the roles of many core effectors remain incompletely understood (Thapa et al. 2020). In this study, we focused on the core effector SDE525, aiming to identify its host targets and elucidate its underlying molecular mechanisms, thereby advancing our understanding of the *C*Las–citrus interaction.

We first confirmed that SDE525 is a secreted protein with a nonspecific subcellular localization pattern, consistent with previous reports (Pitino et al. 2016). Although its transient expression in *N. benthamiana* induced ROS accumulation, it did not trigger a full HR (Mittler et al. 2022). This suggests that SDE525 activates a moderate defence response, which may be insufficient to severely inhibit *C*Las survival and thus represents a finely tuned manipulation of host immunity. Through a combination of Y2H, pull‐down, BiFC, and LCI assays, we identified and validated the host chaperone protein NACα as a direct interacting partner of SDE525. Notably, GO annotations had previously implicated SDE525 in protein folding, a function that aligns with its interaction with a chaperone (Wu et al. 2021).

The functional consequences of this interaction were further investigated. Transient expression of *SDE525* and *NACα* in sweet orange significantly enhanced resistance to *X. citri*, the causal agent of citrus canker. This finding prompted us to hypothesize that SDE525 modulates host defence pathways by targeting NACα. Importantly, we emphasize that this enhanced resistance to a distinct pathogen does not imply a defensive role for SDE525 during authentic *C*Las infection; rather, it represents a paradoxical byproduct of the immune reprogramming required for *C*Las virulence. Intriguingly, this type of inadvertent cross‐protection has been previously observed for other *C*Las effectors, such as AGH17470, whose transient expression similarly enhances citrus resistance to canker (Du et al. 2022). To conclusively validate the SA/JA reprogramming suggested by our untargeted metabolomics—and to address the limitations of less specific antibody‐based methods—we employed targeted HPLC. This robust analytical approach provided definitive, quantitative evidence by precisely distinguishing between free acids and their methylated derivatives. The HPLC data demonstrated that transient expression of SDE525 significantly elevated free SA and MeSA, while simultaneously suppressing free JA and MeJA. Interestingly, manipulation of NACα alone was sufficient to increase SA and decrease JA, but it did not significantly alter MeSA or MeJA levels. These precise quantitative profiles establish that while NACα is the primary driver of the core SA/JA antagonism, SDE525 exerts a broader metabolic influence, extending the hormonal signal via the production of volatile MeSA and the suppression of MeJA.

Turning to the mechanistic basis of the observed JA suppression, our transcriptomic data revealed an apparent paradox: the key JA biosynthetic gene *LOX* was upregulated, while endogenous JA levels decreased. To reconcile this, we propose a strictly hypothetical working model. In this model, the substantial upregulation of *HPL* (hydroperoxide lyase) is hypothesized to divert the common substrate from the JA biosynthetic pathway toward the oxylipin branch, while the strong induction of *JOX2* (jasmonate‐induced oxygenase 2) would accelerate JA catabolism. While this substrate diversion and enhanced catabolism model is mechanistically plausible and fits our transcriptional and hormonal data, we emphasize that it remains a proposed working model. The direct biochemical link between NACα and the transcriptional regulation of *HPL* and *JOX2* is currently speculative and requires further empirical validation.

Firmly grounded in our experimental evidence, we conclude that the *C*Las effector SDE525 targets the host chaperone NACα to reprogramme hormonal homeostasis, specifically activating the SA pathway while suppressing JA accumulation. This establishes the SDE525‐NACα module as a pivotal node in the antagonistic crosstalk between SA and JA signalling, with NACα itself emerging as a promising molecular target for engineering disease resistance.

Finally, we address the broader ecological context of SDE525, strictly distinguishing here between our experimentally supported data and subsequent ecological inferences. Our data conclusively demonstrate that SDE525 suppresses JA—a hormone central to antiherbivore defence. Furthermore, it is an established observation that SDE525 is expressed at significantly higher levels in psyllids than in citrus plants (Thapa et al. 2020). Building on these two discrete lines of evidence, we cautiously speculate that the primary biological function of SDE525 in the *C*Las lifecycle may extend beyond modulating interactions with bacterial pathogens like *X. citri* . We hypothesize that by exploiting NACα to suppress JA, *C*Las may weaken the plant's antiherbivore defences, thereby facilitating its own acquisition and spread by the ACP vector (Figure S6). While this tripartite ecological interpretation is highly intriguing, it remains untested speculation. Future research directly measuring ACP feeding behaviour and performance on plants manipulated for SDE525‐NACα expression will be necessary to validate this hypothesis.

Despite the robust validation provided by our targeted HPLC analysis, we must acknowledge a methodological limitation regarding our multi‐omics integration. There was a discrepancy in the number of biological replicates between the transcriptomic and untargeted metabolomic analyses, which inherently reduces the statistical power of joint pathway enrichment analyses. While our key regulatory pathways (such as α‐linolenic acid and phenylpropanoid biosynthesis) showed consistent enrichment, and the targeted HPLC successfully confirmed the ultimate hormonal outcomes, the specific intermediate metabolic trends derived solely from the untargeted metabolomics should be interpreted with caution. To resolve these limitations and rigorously test our proposed biological models, future studies should optimize multi‐omics integration using advanced statistical frameworks (e.g., Bayesian models) and, most importantly, employ direct molecular approaches—such as yeast one‐hybrid, electrophoretic mobility shift assays, or chromatin immunoprecipitation—to definitively establish whether NACα directly transcriptionally regulates *HPL* and *JOX2* to execute JA catabolism. Additionally, future structural and biochemical studies are required to elucidate exactly how SDE525 binding alters the chaperone activity or client‐protein targeting capacity of CsNACα. Furthermore, to transition our findings from transient expression systems to the context of authentic, long‐term disease, future studies generating stable transgenic citrus lines with tissue‐specific or inducible expression of SDE525 will be essential to validate these findings under long‐term *C*Las infection pressure.

## 4 Experimental Procedures

### 4.1 Plant Materials and Microbial Strains

*Nicotiana benthamiana* plants were grown in a controlled greenhouse at 28°C under a 16 h light/8 h dark photoperiod. Sweet orange ( *C. sinensis* ‘Newhall’) seedlings were maintained in a separate greenhouse at a constant temperature of 28°C. *E. coli* DH5α and BL21 (DE3) (Weidibio Co. Ltd.) were used for cloning and protein expression, respectively. *Agrobacterium tumefaciens* GV3101 (Weidibio Co. Ltd.) was used for all transient expression assays. *E. coli* was cultured in Luria Bertani (LB) medium at 37°C, while *A. tumefaciens* was grown in LB medium at 28°C. For long‐term storage, bacterial cultures were preserved at −80°C in LB medium supplemented with 60% (v/v) glycerol. When required, antibiotics were added to the medium at the following final concentrations: kanamycin, 50 μg/mL; and rifampicin, 20 μg/mL.

### 4.2 Plasmid Construction

All plasmids used in this study are listed in Table S1, and the corresponding primers are listed in Table S2. Plasmids were constructed using the In‐Fusion Snap Assembly Kit (Takara Bio) following the manufacturer's instructions for homologous recombination. The sequences of all constructed plasmids were confirmed by Sanger sequencing (Tsingke Biotechnology).

### 4.3 PhoA Assay

A PhoA assay was performed following a previously described method (Zhang et al. 2020), to determine whether SDE525 is a secreted protein. The mature PhoA (lacking its native signal peptide), termed mPhoA, was cloned into the pET30a vector to generate pET30a‐T7‐mPhoA, which serves as a negative control. The coding sequence of SDE525, with or without its N‐terminal signal peptide (SP), was fused in‐frame to the N‐terminus of mPhoA (the version without the SP was named m00525). This created four constructs: pET30a‐T7‐SP‐mPhoA (positive control), pET30a‐T7‐mPhoA (negative control), pET30a‐T7‐SDE525‐mPhoA (test construct), and pET30a‐T7‐m00525‐mPhoA (test construct).

The resulting plasmids were first transformed into *E. coli* DH5α for propagation, and positive clones were confirmed by colony PCR. Subsequently, the verified plasmids were transformed into *E. coli* BL21 (DE3) for protein expression. Transformants were streaked onto LB agar indicator plates supplemented with 90 μg/mL 5‐bromo‐4‐chloro‐3‐indolyl phosphate (BCIP), 100 mM isopropyl‐β‐D‐thiogalactopyranoside (IPTG), and 100 mM Na_2_HPO_4_. After incubation at 37°C, the development of blue colouration indicated functional PhoA activity in the periplasm, signifying that the fused N‐terminal sequence possesses a functional signal peptide for Sec‐dependent secretion. Conversely, white colonies indicated a lack of secretion.

### 4.4 *Agrobacterium*‐Mediated PVX Infection Assay

SDE525 and BAX were amplified with the corresponding primers shown in Table S2 and cloned into the PVX‐based binary plant vector pGR106 (Du et al. 2022). The recombinant vectors were introduced into *A. tumefaciens* GV3101. PVXm00525, PVX‐BAX, PVX, and buffer were first infiltrated into four‐ to six‐leaf stage *N. benthamiana* leaves. At 72 hpi, a portion of the infiltrated leaves was removed for histochemical staining with 3,3′‐diaminobenzidine (Vanacker et al. 2000; Zhang et al. 2023). The infiltrated leaves were collected to perform electrolyte leakage measurement 3–7 days after inoculation, as previously described (Pitino et al. 2016; Zhang et al. 2019).

### 4.5 RNA Isolation and RT‐qPCR Analysis

Total RNA was extracted by using a plant RNA isolation kit (TransGen Biotech). Total RNA was reverse transcribed using EasyScript All‐in‐One First‐Strand cDNA Synthesis Super Mix for qPCR (TransGen Biotech). qPCR assays were conducted with TransStart Top Green qPCR SuperMix (TransGen Biotech). Relative expression values were calculated using the 2^−ΔΔCt^ method with defence responses gene of plants, *NbEF1α* of *N. benthamiana*, *CsActin* of *C. sinensis* as reference genes (Dai et al. 2023).

### 4.6 *Agrobacterium tumefaciens* ‐Mediated Transient Expression and Fluorescence Observation

For subcellular localization and protein–protein interaction assays, *A. tumefaciens* GV3101 strains harbouring the binary vectors pC1300‐35S‐eGFP, pC1300‐35S‐eGFP‐m00525, pC1300‐35S‐eGFP‐NACα, pC1300‐35S‐mCherry, and pC1300‐35S‐mCherry‐m00525 were cultured overnight. Bacterial cells were harvested by centrifugation and resuspended in an infiltration buffer (10 mM 2‐(*N*‐morpholino)ethanesulfonic acid [MES], 10 mM MgCl_2_, 200 μM acetosyringone, pH 5.6) (Pang et al. 2020). The suspensions were adjusted to a final optical density at 600 nm (OD_600_) of 1.0 and infiltrated into the abaxial side of fully expanded *N. benthamiana* leaves using a 1 mL needleless syringe. At 2 days post‐infiltration (dpi), fluorescence signals were visualized using a laser scanning confocal microscope (LSM series, Zeiss). The detection parameters were set as follows: eGFP was excited at 488 nm, with emission captured between 500 and 530 nm; mCherry was excited at 559 nm, with emission captured between 595 and 625 nm (Zhou et al. 2020).

### 4.7 Immunoprecipitation Assay

The recombinant vectors pC1300‐35S‐eGFP, pC1300‐35S‐eGFP00525 were introduced into *A. tumefaciens* GV3101 (P19). The pC1300‐35S‐eGFP transformed *A. tumefaciens* was infiltrated into *C. sinensis* leaves. Following a 48 h incubation period post‐infiltration, leaves were harvested and total proteins were extracted using a standardized protein extraction buffer. The extracted total protein was then incubated with GFP beads (Sigma) to capture GFP‐tagged protein complexes. The eluted protein complexes were subsequently subjected to LC–MS/MS analysis at Shanghai Majorbio Bio‐pharm Technology Co. Ltd., aimed at identifying potential citrus protein targets (Basu et al. 2022).

### 4.8 Y2H Assays

The coding regions of SDE525 were amplified and inserted into the EcoRI and BamHI sites of vector pGBKT7. Full‐length cDNAs of *CsNACα* (*CISIN_1g028148mg*) were amplified and cloned into pGADT7 vectors. AD‐CsNACα was co‐transformed with BK‐m00525 in yeast to confirm the interaction (Ying et al. 2019).

### 4.9 Pull‐Down Assays

The plasmids pET32a‐m00525, pGEX‐6T‐1, and pGEX‐6 T‐1‐NACα were transformed into *E. coli* BL21 (DE3) cells, and *E. coli* transformants were grown in LB broth at 37°C for 14 h. These transformants were then subcultured into 100 mL LB medium and allowed to grow until OD_600_ = 0.6, then isopropyl‐β‐D‐thiogalactopyranoside (IPTG) was added to a final concentration of 1 mM to express target proteins at 18°C for 24 hpi. Cells were then collected and lysed by a JY92‐IIN ultrasonic cell‐crushing instrument (GIPP) for GST, GST‐tagged, and His‐tagged proteins extraction. An appropriate amount of total lysates was used as input controls. His‐tagged 00525 proteins were individually purified by His‐tag Purification Resin (Shenggong), GST and GST‐NACα proteins were incubated with immobilized His‐tagged 00525 at 4°C for 3 h. The mixtures were washed three times in wash buffer (phosphate‐buffered saline containing Tween 20) and eluted the proteins from the resin beads by boiling in 1 × SDS sample buffer for 10 min. Subsequently, these proteins were identified by western blot analysis using anti‐GST (Abmart) and anti‐His (Abmart) antibodies. An anti‐mouse antibody (Shenggong) was used as the secondary antibody (Jiang et al. 2025).

### 4.10 BiFC Assay

M00525 was inserted into pC1300‐nYFP using the primer pair pC1300‐nYFP F/R to generate pC1300‐35S‐nYFP‐m00525. The cDNA of CsNACα was inserted into pC1300‐35S‐cYFP using the primer pair pC1300‐35S‐cYFP F/R to generate pC1300‐35S‐cYFP‐CsNACα. The pC1300‐35S‐nYFP‐m00525 and pC1300‐35S‐cYFP‐CsNACα were co‐transformed into *A. tumefaciens* GV3101 and then cultured in LB medium separately overnight. The *Agrobacterium* cultures were centrifuged, resuspended in induction buffer (100 mM MES, 10 mM MgCl_2_, 2 mM acetosyringone) with OD_600_ = 0.8–1.0, equal volume mixed, and incubated for 3 h at 25°C (Oh et al. 2022). The buffer containing *A. tumefaciens* was infiltrated into the lower epidermis of *N. benthamiana* plants. Combinations of pC1300‐35S‐nYFP‐m00525 + pC1300‐35S‐cYFP and pC1300‐35S‐cYFP‐CsNACα + pC1300‐35S‐nYFP served as controls.

*Agrobacterium tumefaciens* GV3101 carrying pC1300‐35S‐Yn and pC1300‐35S‐Yc derivatives were mixed in a 1:1 ratio at the final OD_600_ value of 0.4. Fluorescence was observed at 2 dpi using a laser scanning confocal microscope, with 488 nm excitation.

### 4.11 LCI Assay

M00525 was inserted into pC1300‐35S‐nLUC using the primer pair pC1300‐35S‐nLUC F/R to generate pC1300‐35S‐nLUC‐m00525. The CsNACα was inserted into pC1300‐35S‐cLUC using the primer pair pC1300‐35S‐cLUC F/R to generate pC1300‐35S‐cLUC‐CsNACα. The pC1300‐35S‐nLUC‐m00525 and pC1300‐35S‐cLUC‐CsNACα were co‐transformed into *A. tumefaciens* GV3101 and then cultured in LB medium separately overnight. The inoculation method was as previously described. The abaxial leaf surface was sprayed with 1 mM D‐luciferin substrate solution, followed by a 7‐min dark adaptation period for the plants. The luminescence images were captured using a CCD imaging system (Shi et al. 2025).

### 4.12 Assay of SA and JA Levels

Determination of endogenous SA and JA levels in citrus leaves with transient expression of the m00525 and NACα genes via ELISA (Chen et al. 2021). Young citrus leaves (4–6 weeks old) were inoculated with *A. tumefaciens* strains transformed with pC1300‐35S‐eGFP, pC1300‐35S‐eGFP‐m00525 and pC1300‐35S‐eGFP‐NACα. Samples were collected 3 days post‐inoculation for the determination of SA and JA contents.

### 4.13 *Xanthomonas citri* Infiltration Assays

The overexpression constructs were transformed into *A. tumefaciens* GV3101. Citrus transient expression and *X. citri* infiltration assays were conducted as previously described. To evaluate disease susceptibility, sweet orange ( *C. sinensis* ) leaves were first subjected to transient expression. After a 7–14‐day incubation period to ensure transgene expression, the leaves were gently punctured with a sterile syringe needle at the agroinfiltration site (Du et al. 2022; Du et al. 2023). A suspension of *X. citri* was then infiltrated into the wounds. Disease symptoms were subsequently observed and photographed (Yan et al. 2023). Three biological replicates were performed for each treatment. Leaf lesions were punched for sampling, followed by homogenization in sterile water. The resulting suspensions were serially diluted and plated. Colony‐forming units (CFUs) were counted to determine microbial titres.

### 4.14 Transcriptome and Metabolome Sequencing

To investigate the defence response signalling pathways targeted by m00525 and NACα, transient expression of pC1300‐35S‐eGFP‐m00525, pC1300‐35S‐eGFP‐CsNACα, and pC1300‐eGFP was performed in citrus for 3 days, followed by transcriptome and metabolome sequencing at General Biol (Anhui) Co. Ltd.

### 4.15 Plant Hormone Determination

For free SA and MeSA, ground leaf samples were extracted twice with 90% methanol at 4°C. After centrifugation (12,000 *g*, 10 min), the combined supernatants were evaporated to dryness under nitrogen at 40°C. The residue was reconstituted in 0.5 mL of water with 50 μL of trichloroacetic acid (1 mg/mL) and extracted twice with ethyl acetate/cyclohexane (1:1, v/v). The combined organic phases were dried under nitrogen, redissolved in 0.3 mL of pure methanol, and filtered through a 0.22‐μm microporous membrane. The free SA and MeSA contents were analysed on an HPLC system using a C18 column (5 μm, 4.6 × 250 mm) at a flow rate of 0.8 mL/min and column temperature of 30°C. Detection was monitored at 302 nm using a PDA detector. The mobile phase was methanol:1% acetic acid aqueous solution (60:40 by volume). The injection volume was 10 μL. Free SA and MeSA were determined using the external standard method.

For free JA and MeJA, leaf samples were ground in an ice bath with 1 mL of 60% methanol‐acetic acid aqueous solution and extracted using a 20‐min ice‐water ultrasonic treatment. After centrifugation at 12,000 *g* for 10 min, the supernatant was collected. The pellet was re‐extracted twice with 60% methanol‐acetic acid aqueous solution. The combined supernatants were evaporated to dryness under nitrogen, reconstituted in 0.5 mL of pure methanol, and filtered through a 0.22‐μm microporous membrane. The JA and MeJA contents were analysed on an HPLC system using a C18 column (5 μm, 4.6 × 250 mm) at a flow rate of 1.0 mL/min and column temperature of 30°C. Detection was monitored at 210 nm. The mobile phase was methanol‐acetonitrile (1:1) and 0.01% aqueous phosphoric acid (60:40 by volume). The injection volume was 10 μL. JA and MeJA were determined using the external standard method.

### 4.16 Statistical Analysis

All statistical analyses were performed using SPSS v. 20 software. Data are presented as the mean ± standard deviation (SD). Biological replicates were defined as independent biological samples (e.g., independent plants or independently treated leaf samples), while technical replicates referred to repeated measurements of the same biological sample. For phenotypic assessments, RT‐qPCR validation, and HPLC‐based hormone quantification, each experiment contained at least three independent biological replicates. Differences between two groups were evaluated using a two‐tailed Student's *t*‐test (*p < 0.05, **p < 0.01, ***p < 0.001, ****p < 0.0001). Comparisons among multiple groups were analysed using one‐way ANOVA followed by Duncan's multiple range test (*p* < 0.05), and significant differences are indicated by different lowercase letters.

For transcriptomic and metabolomic analyses, raw data were normalized using FPKM for RNA‐seq, and total ion current normalization for metabolomics. DEGs and DAMs were identified using DESeq2. The thresholds for determining significant differences were set at a false discovery rate (FDR) adjusted *p* < 0.05 and an absolute fold change log_2_FC > |1|.

## Author Contributions

**Yijie Zhan:** conceptualization, methodology, software, validation, formal analysis, visualization, writing – review and editing, data curation. **Zixiang Sun:** investigation, formal analysis, software, data curation, validation. **Changwei Luo:** data curation, formal analysis, investigation, validation. **Zehua Zhou:** methodology, conceptualization, data curation, supervision, writing – review and editing, validation, formal analysis. **Na Song:** supervision, validation, data curation, methodology. **Jinxing Liao:** methodology, data curation, validation, formal analysis, supervision. **Jiefu Deng:** conceptualization, methodology, software, data curation, investigation, validation, formal analysis, visualization, funding acquisition, writing – original draft. **Tuyong Yi:** project administration, resources, writing – review and editing, funding acquisition, visualization, supervision, methodology.

## Funding

This work was supported by the National Key Research and Development Program of China, 2021YFD1400800. the Hunan Provincial Key R&D Program, 2022NK2052. Hunan Agriculture Research System, HARS‐09. Hunan Province Postgraduate Research and Innovation Project, CX20240644.

## Conflicts of Interest

The authors declare no conflicts of interest.

## Supporting information


**Figure S1:** Western blotting of anti‐GFP magnetic bead elution protein solution.

**Figure S2:** Co‐localization of m00525 with NACα.

**Figure S3:** Differentially expressed gene numbers of the three comparison groups.

**Figure S4:** Comparative transcriptome and metabolome analysis between the three comparison groups.

**Figure S5:** Expression patterns of the selected 10 differentially expressed genes (DEGs) and 2 differentially accumulated metaboilites (DAMs).

**Figure S6:** The effect of ‘*Candidatus* Liberibacter asiaticus’ effector CLIBASIA_00525 on the citrus immune due to the target proteins.

**Table S1:** Restriction enzyme cutting site.

**Table S2:** Primer sequence.

**Table S3:** Transcriptome Data Statistics.

**Table S4:** Summary of differentially expressed genes (DEGs) across three comparison groups: Control versus m00525, Control versus NACα, and m00525 versus NACα.

**Table S5:** List of differentially expressed genes (DEGs) involved in jasmonic acid (JA)/salicylic acid (SA) signalling and plant defence responses.

## Data Availability

The data that support the findings of this study are available from the corresponding author upon reasonable request.
