# Supplementary material for: ‘Candidatus Liberibacter asiaticus’ Effector SDE525 hijacks NACα to Suppress Jasmonic Acid‐Mediated Immunity in Citrus
Source: Mol Plant Pathol. 2026 May 18;27(5):e70272. doi: 10.1111/mpp.70272 (PMC13181327; doi:10.1111/mpp.70272)
Supplement: Supplementary file 1 — Figure S1: Western blotting of anti‐GFP magnetic bead elution protein solution. [file MPP-27-e70272-s003.docx]

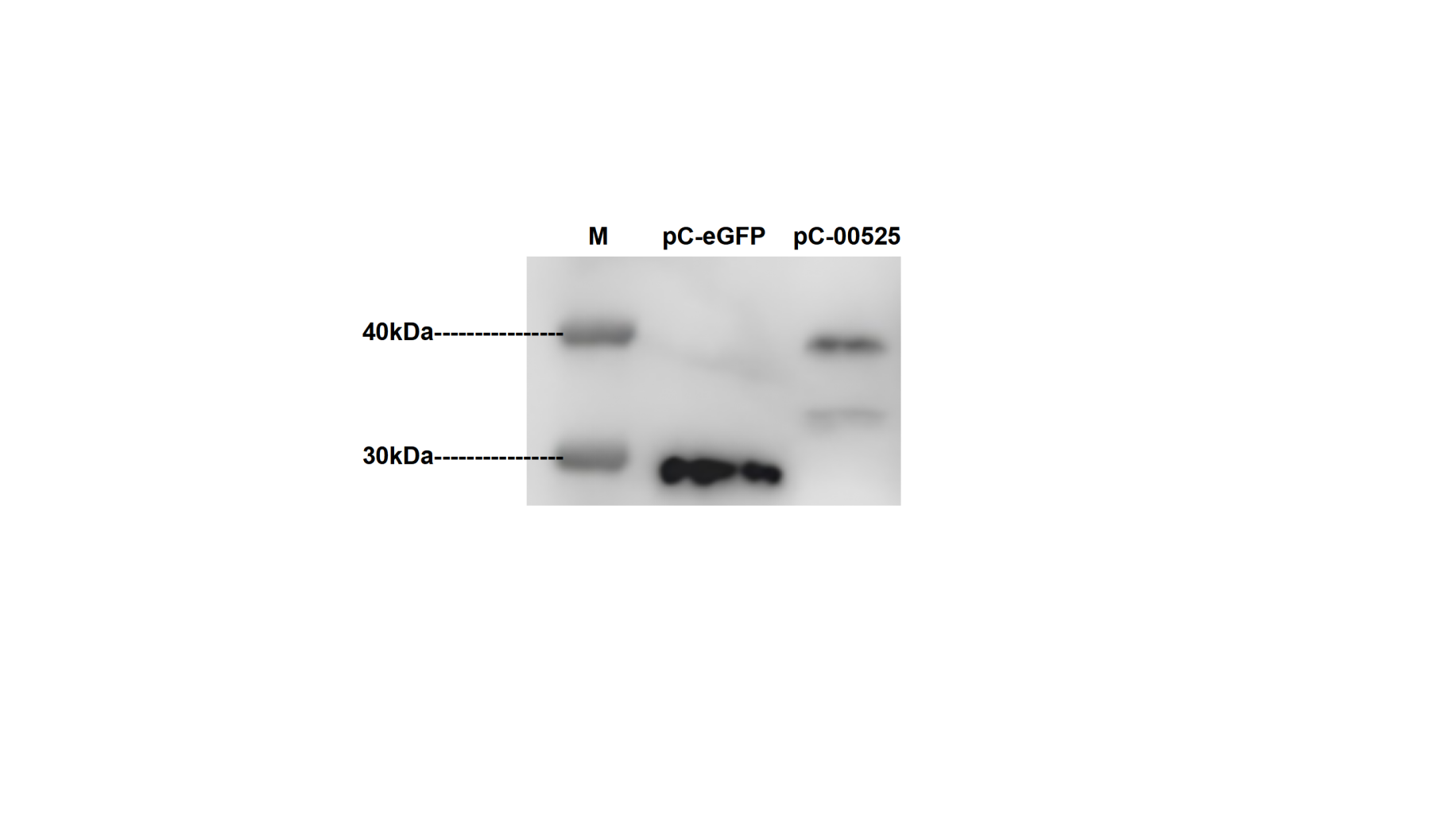


**Supplementary Figure S1.** Western blotting of anti-GFP magnetic bead elution protein solution.The blot was probed with an anti-GFP antibody (Proteintech Group, Inc).M, molecular marker.
