# Supplementary material for: ‘Candidatus Liberibacter asiaticus’ Effector SDE525 hijacks NACα to Suppress Jasmonic Acid‐Mediated Immunity in Citrus
Source: Mol Plant Pathol. 2026 May 18;27(5):e70272. doi: 10.1111/mpp.70272 (PMC13181327; doi:10.1111/mpp.70272)
Supplement: Supplementary file 2 — Figure S2: Co‐localization of m00525 with NACα. [file MPP-27-e70272-s005.docx]

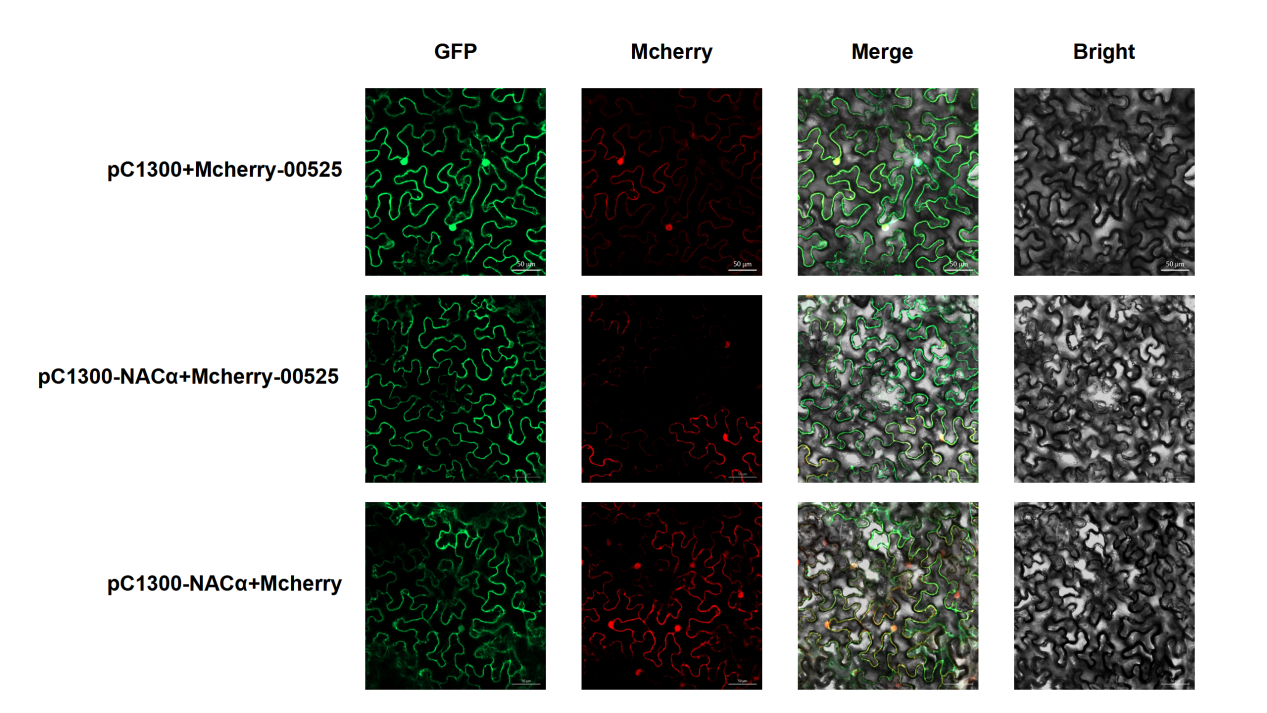


**Supplementary Figure S2** Co-localization of m00525 with NACα. Images were taken using confocal microscopy. Scale bars, 50 μm.
