# Supplementary figures and images for: ‘Candidatus Liberibacter asiaticus’ Effector SDE525 hijacks NACα to Suppress Jasmonic Acid‐Mediated Immunity in Citrus
Source: Mol Plant Pathol. 2026 May 18;27(5):e70272. doi: 10.1111/mpp.70272 (PMC13181327; doi:10.1111/mpp.70272)

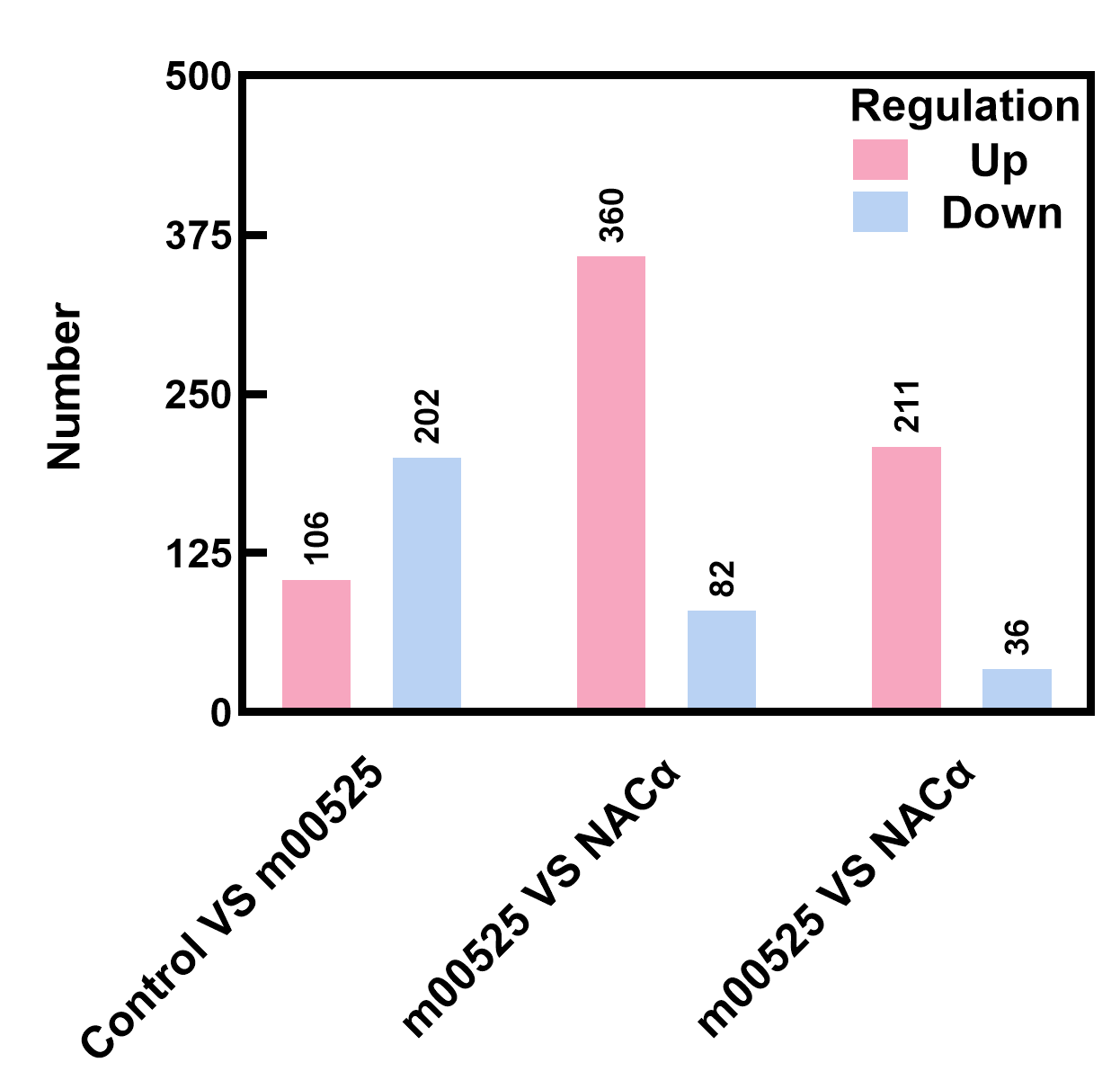


**Supplementary Figure S3.** DEGs’ number of the three comparison groups.

Supplement: Supplementary file 3 — Figure S3: Differentially expressed gene numbers of the three comparison groups. [file MPP-27-e70272-s011.docx]
