# Supplementary material for: ‘Candidatus Liberibacter asiaticus’ Effector SDE525 hijacks NACα to Suppress Jasmonic Acid‐Mediated Immunity in Citrus
Source: Mol Plant Pathol. 2026 May 18;27(5):e70272. doi: 10.1111/mpp.70272 (PMC13181327; doi:10.1111/mpp.70272)
Supplement: Supplementary file 4 — Figure S4: Comparative transcriptome and metabolome analysis between the three comparison groups. [file MPP-27-e70272-s009.docx]

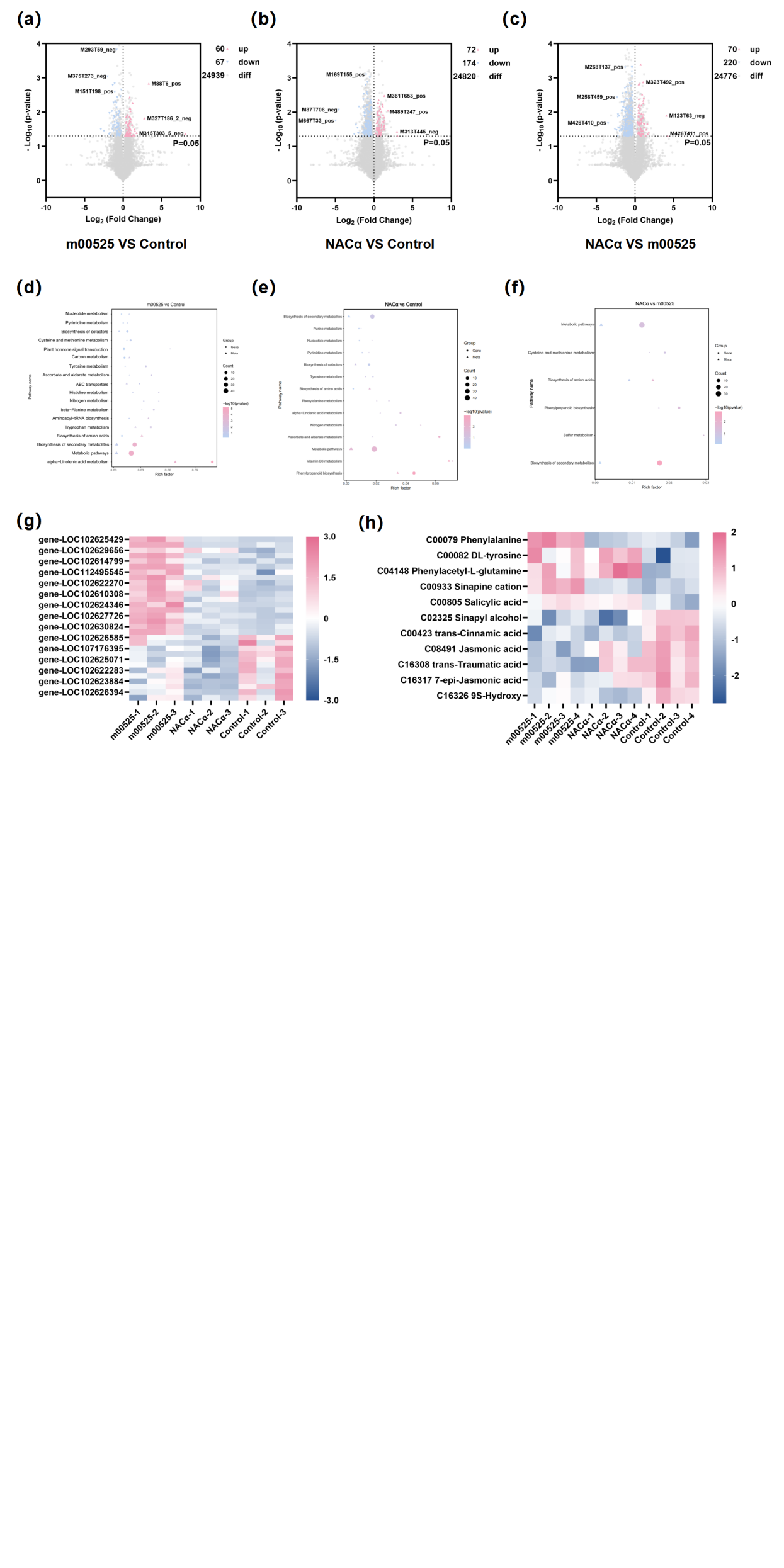


**Supplementary Figure S4.** Comparative transcriptome and metabolome analysis between the three comparison groups. **(a-c).** The differential metabolites expression analysis showing up-and down-regulated genes across all three comparison groups. A log_2_FC > 1 , P Value < 0.01 and VIP(Variable Importance in Projection)> 1 was indicated as significantly up-regulated in red, while a log_2_FC < -1 and P Value < 0.01 and VIP(Variable Importance in Projection)> 1was indicated as significantly down-regulated in blue. **(d-f).** The bubble plot illustrates the KEGG pathways that were significantly co-enriched in both the transcriptome and metabolome for each pairwise comparison of treatments. **(g-h).** The heatmap displays the expression profiles of DEGs and the DAMs involved in the alpha-linolenic acid metabolism and phenylpropanoid biosynthesis pathways.
