# Supplementary material for: ‘Candidatus Liberibacter asiaticus’ Effector SDE525 hijacks NACα to Suppress Jasmonic Acid‐Mediated Immunity in Citrus
Source: Mol Plant Pathol. 2026 May 18;27(5):e70272. doi: 10.1111/mpp.70272 (PMC13181327; doi:10.1111/mpp.70272)
Supplement: Supplementary file 5 — Figure S5: Expression patterns of the selected 10 differentially expressed genes (DEGs) and 2 differentially accumulated metaboilites (DAMs). [file MPP-27-e70272-s002.docx]

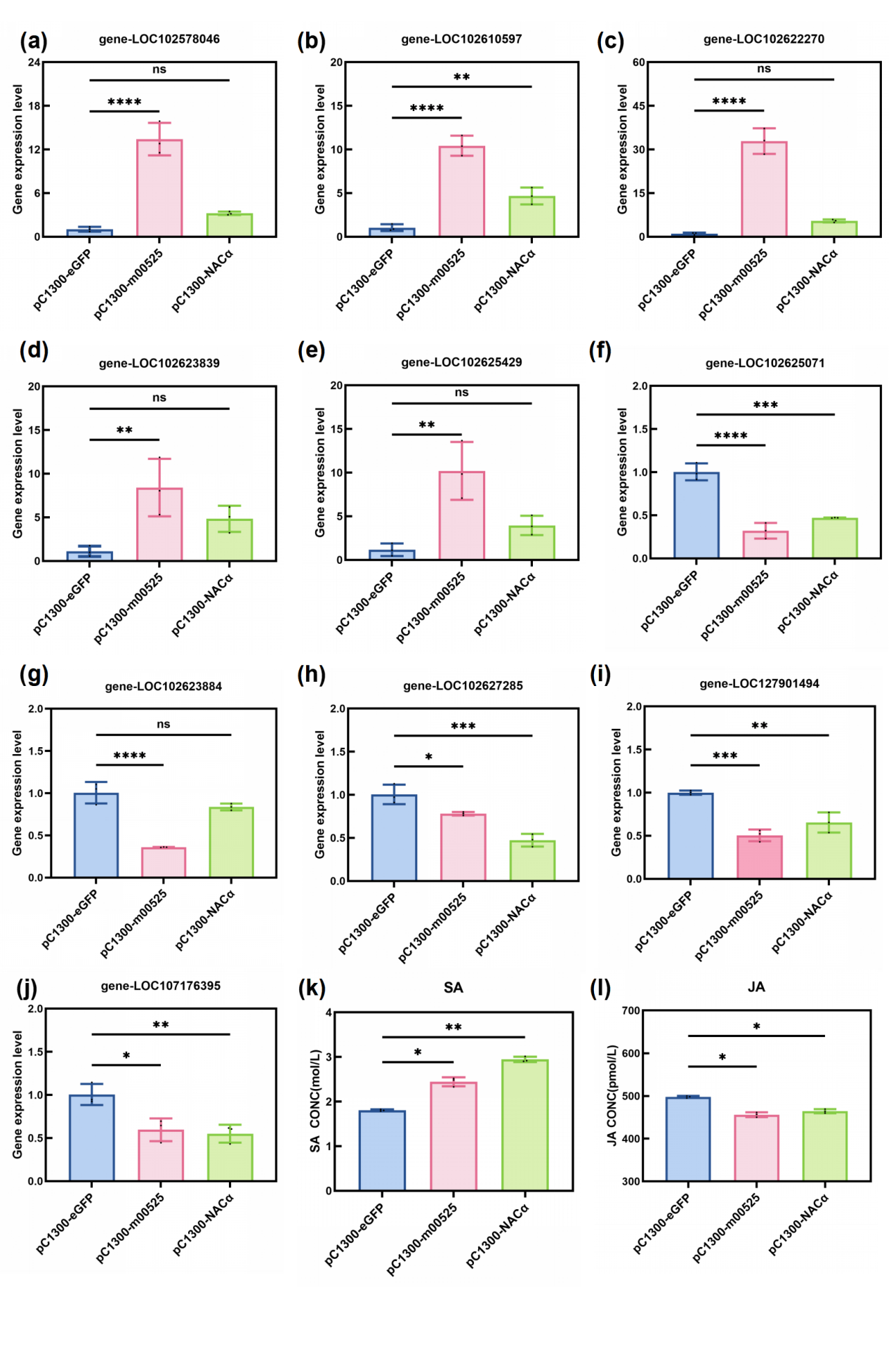


**Supplementary Figure S5.** Expression patterns of the selected 10 DEGs and 2 DAMs.RT-qPCR analysis of transcript levels of ten DEGs. The mRNA abundance of each indicated gene was normalized to the expression of *CsActin*.

*CsLOX2.1* (gene-LOC102625429), *CsJOX2* (gene-LOC102610597), *CsADHL* (gene-LOC102623839), *CsHPL* (gene-LOC102578046), *CsKTI* (gene-LOC102622270), *CsLECRK2* (gene-LOC102625071), *CsF-BOX* (gene-LOC102623884), *CsLECRK3*

(gene-LOC102627285), *CsRSR1* (gene-LOC127901494), gene-LOC107176395. Elisa analysis of Metabolic level of twe DAMs.Data are presented as mean ± SD (*n* = 3), and asterisks indicate significant differences (Student’s *t*-test, *, *P* < 0.05; **, *P* < 0.01; ***, *P* < 0.001).
