# Supplementary material for: ‘Candidatus Liberibacter asiaticus’ Effector SDE525 hijacks NACα to Suppress Jasmonic Acid‐Mediated Immunity in Citrus
Source: Mol Plant Pathol. 2026 May 18;27(5):e70272. doi: 10.1111/mpp.70272 (PMC13181327; doi:10.1111/mpp.70272)
Supplement: Supplementary file 6 — Figure S6: The effect of ‘Candidatus Liberibacter asiaticus’ effector CLIBASIA_00525 on the citrus immune due to the target proteins. [file MPP-27-e70272-s006.docx]

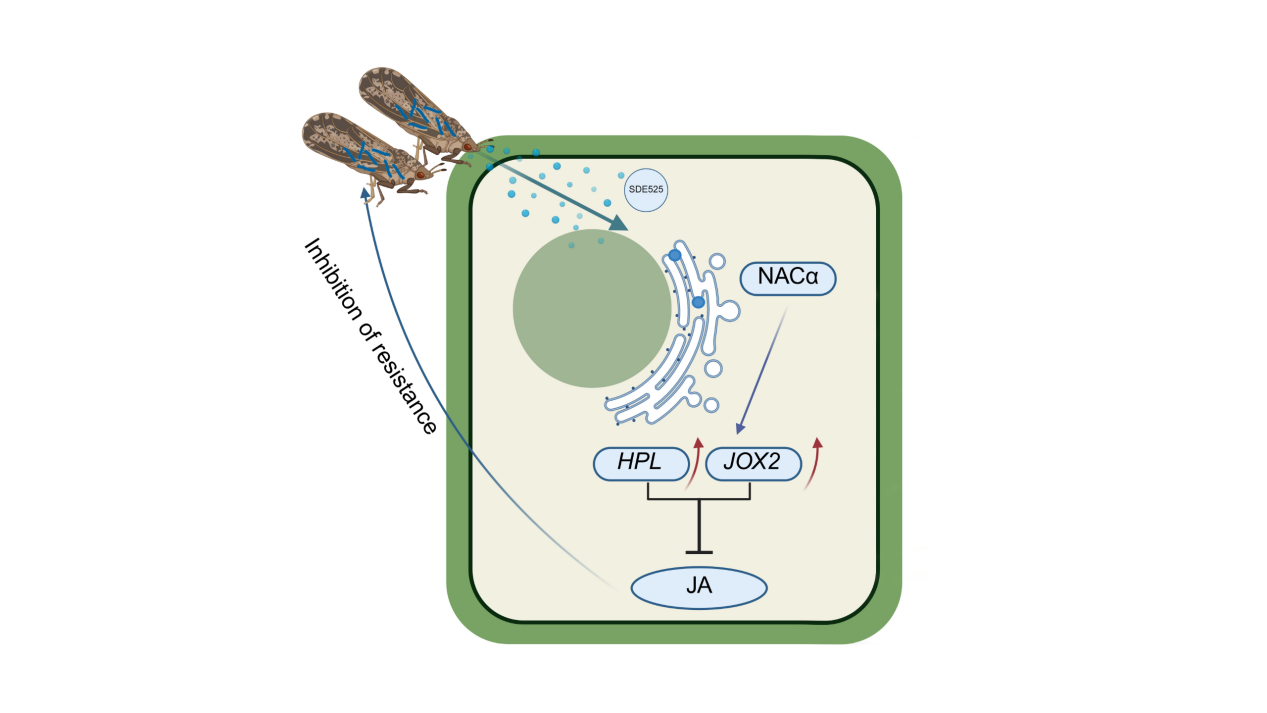


**Supplementary Figure S6.** The effect of *C*Las effector CLIBASIA_00525 on the citrus immune due to the target proteins.
