# Supplementary material for: ‘Candidatus Liberibacter asiaticus’ Effector SDE525 hijacks NACα to Suppress Jasmonic Acid‐Mediated Immunity in Citrus
Source: Mol Plant Pathol. 2026 May 18;27(5):e70272. doi: 10.1111/mpp.70272 (PMC13181327; doi:10.1111/mpp.70272)
Supplement: Supplementary file 7 — Table S1: Restriction enzyme cutting site. [file MPP-27-e70272-s010.docx]

**Supplement Table S1.** Restriction enzyme cutting site.

| Vectors name | Restriction enzyme cutting site |
| --- | --- |
| pET30a-T7 | KpnⅠ/HindⅢ |
| pGR106-35S | SalⅠ/AscⅠ |
| pCAMIBIA1300-35S-EGFP | KpnⅠ/SalⅠ |
| pCAMIBIA1300-35S-mCherry | KpnⅠ/BamHⅠ |
| pCAMIBIA1300-35S-NYFP | SpeⅠ/SacⅠ |
| pCAMIBIA1300-35S-CYFP | SpeⅠ/KpnⅠ |
| pCAMIBIA1300-35S-nLUC | BamHⅠ/SalⅠ |
| pCAMIBIA1300-35S-cLUC | BamHⅠ/SalⅠ |
| pET32a-T7 | BamHⅠ/XhoⅠ |
| pGEX-6P-1-TAC | BamHⅠ/XhoⅠ |
| pGADT7 | EcoRⅠ/BamHⅠ |
| pGBKT7 | EcoRⅠ/SalⅠ |
