# Supplementary material for: ‘Candidatus Liberibacter asiaticus’ Effector SDE525 hijacks NACα to Suppress Jasmonic Acid‐Mediated Immunity in Citrus
Source: Mol Plant Pathol. 2026 May 18;27(5):e70272. doi: 10.1111/mpp.70272 (PMC13181327; doi:10.1111/mpp.70272)
Supplement: Supplementary file 8 — Table S2: Primer sequence. [file MPP-27-e70272-s008.docx]

**Supplement Table S2.** Primer sequence.

| Primer name | Primer sequence |
| --- | --- |
| pET32a-00525/F | GGCTGATATCGGATCATGTGTTCTGATTATGTATATGAAG |
| pET32a-00525/R | TGCTCGAGTGCGGCCGTCTCTTTCTCATTTTGCTCAATG |
| pET30a-00525/F | ACAGCCCAGATCTGGATGTGTTCTGATTATGTATATGAAG |
| pET30a-00525/R | GTGCGGCCGCAAGCTTCTCTTTCTCATTTTGCTCAATGG |
| pGR106-00525/F | GCATCGATTGGCGCGCCATGTGTTCTGATTATGTATATGAAG |
| pGR106-00525/R | GCGGTCGACGCGGCCGCTTACTCTTTCTCATTTTGCTCAATG |
| pC1300-00525/F | CGGGGGACGAGCTCGGTACCATGTGTTCTGATTATGTATATGAAG |
| pC1300-00525/R | ACGAGATCTGGTCGACCTCTTTCTCATTTTGCTCAATGG |
| pC1300mcherry-00525/F | CGGGGGACGAGCTCGGTACCATGTGTTCTGATTATGTATATGAAG |
| pC1300mcherry-00525/R | TGCTCACCATGGATCCCTCTTTCTCATTTTGCTCAATGG |
| pC1300NY-00525/F | GATCGGGGAAATTCGAGCTCTTACTCTTTCTCATTTTGCTCAATG |
| pC1300NY-00525/R | GCCCAGGCCTACTAGTTGTTCTGATTATGTATATGAAGATG |
| pC1300CY-NACα/R | GATCGGGGAAATTCGAGCTCTCAAGTAGTAAGCTCCATGATAGCA |
| pC1300CY-NACα/F | GCCCAGGCCTACTAGTATGTCGCCACCAGCTCCC |
| pC1300NL-00525/F | TCGGTACCCGGGATCATGTGTTCTGATTATGTATATGAAG |
| pC1300NL-00525/R | ACGAGATCTGGTCGACCTCTTTCTCATTTTGCTCAATGG |
| pC1300CL-NACα/F | GCGGTACCCGGGATCCCATGTCGCCACCAGCTCC |
| pC1300CL-NACα/R | AGCTCTGCAGGTCGATCAAGTAGTAAGCTCCATGATAGC |
| pGEX-NACα/F | GGGGCCCCTGGGATCCATGTCGCCACCAGCTCCC |
| pGEX-NACα/R | GATGCGGCCGCTCGAGAGTAGTAAGCTCCATGATAGCACT |
| pAD-NACα/F | GGAGGCCAGTGAATTCATGTCGCCACCAGCTCCC |
| pAD-NACα/R | CGAGCTCGATGGATCCAGTAGTAAGCTCCATGATAGCACT |
| pBK-00525/F | CATGGAGGCCGAATTCATGTGTTCTGATTATGTATATGAAG |
| pBK-00525/R | GCCGCTGCAGGTCGACCTCTTTCTCATTTTGCTCAATGG |
