# Supplementary material for: ‘Candidatus Liberibacter asiaticus’ Effector SDE525 hijacks NACα to Suppress Jasmonic Acid‐Mediated Immunity in Citrus
Source: Mol Plant Pathol. 2026 May 18;27(5):e70272. doi: 10.1111/mpp.70272 (PMC13181327; doi:10.1111/mpp.70272)
Supplement: Supplementary file 9 — Table S3: Transcriptome Data Statistics. [file MPP-27-e70272-s004.docx]

**Supplement Table S3.**Transcriptome Data Statistics.

| **Samples** | **Reads** | **Clean reads** | **Uniquely mapped(%)** | **Q30 (%)** | **GC (%)** |
| --- | --- | --- | --- | --- | --- |
| Control-1 | 38520080 | 38399094 | 83.5242 | 95.15 | 43.95 |
| Control-2 | 40021576 | 39878504 | 82.9357 | 95.12 | 44.27 |
| Control-3 | 41335108 | 41227298 | 87.3642 | 95.14 | 43.52 |
| m00525-1 | 38233928 | 38098988 | 85.5281 | 95.34 | 43.68 |
| m00525-2 | 36588644 | 36488144 | 87.5311 | 95.32 | 43.51 |
| m00525-3 | 39744248 | 39648172 | 80.4527 | 95.25 | 44.19 |
| NACα-1 | 43931086 | 43747724 | 87.1923 | 94.94 | 43.84 |
| NACα-2 | 56132540 | 55985776 | 84.9028 | 95.36 | 43.81 |
| NACα-3 | 36607034 | 36499972 | 82.3726 | 95.10 | 44.33 |
